# How Covid Flipped the Classroom: Learning from the Next Generation of Gerontologists

**DOI:** 10.1093/geroni/igaa057.3210

**Published:** 2020-12-16

**Authors:** Mary Carter

**Affiliations:** Towson University, Towson, Maryland, United States

## Abstract

Covid laid bare the structural inequities and systemic challenges facing LTC, both in terms of risk and neglect. Lost in the daily tally of new cases and mortality rates are the stories of essential workers who have neither degree nor stature in the field, yet who fulfill an awesome responsibility—caring for older adults no longer visible behind locked doors. Suddenly, these full-time students and part-time workers found themselves employed in one of the most dangerous occupations in the country—providing direct care in nursing homes and assisted living communities. This award lecture highlights their voices and lessons learned.

